# Seasonal Hydration Status of Common Bryophyte Species in Azorean Native Vegetation

**DOI:** 10.3390/plants12162931

**Published:** 2023-08-14

**Authors:** Márcia C. M. Coelho, Rosalina Gabriel, Claudine Ah-Peng

**Affiliations:** 1 cE3c/GBA—Centre for Ecology, Evolution and Environmental Changes/Azorean Biodiversity Group and CHANGE—Global Change and Sustainability Institute, PT-9700-042 Angra do Heroísmo, Portugal; 2 School of Agricultural and Environmental Sciences, University of the Azores, PT-9700-042 Angra do Heroísmo, Portugal; 3 Pôle de Protection des Plantes, UMR PVBMT, Université de La Réunion, Pôle de Protection des Plantes, 7 Chemin de l’IRAT, 97410 Saint-Pierre, France

**Keywords:** in situ studies, water retention capacity, desiccation tolerance, poikilohydry, life form, climate change, forest, liverworts, mosses, Azores

## Abstract

Bryophytes play a crucial role in the ecosystem’s water compartment due to their unique ability to retain water. However, their role within temperate native ecosystems is mostly unknown. To address this knowledge gap, a study was conducted on Terceira Island (Azores), focusing on 14 bryophyte species found at different altitudes (40 m, 683 m, and 1012 m); five samples were collected monthly, per species and location, and their fresh, saturated, and dry weights were examined in the laboratory; four species were collected from more than one site. Generalized linear models (GLM) were used to assert the influence of climate factors (temperature, precipitation, and relative humidity) and environmental variables on two water indicators: field water content (FWC) and relative water content (RWC). None of the examined factors, per se, were able to explain all cases. Species appear to respond to climate according to a limiting factor effect: at lower elevations, precipitation was determinant, while at medium elevations, FWC was influenced by a combination of precipitation and relative humidity. At higher elevations, temperature was retained for seven of the nine studied species. The RWC values indicated that the 14 bryophyte species remained hydrated throughout the year but rarely reached their maximum water-holding capacity, even at the highest altitude. Understanding the mechanisms by which native bryophytes acquire, store, and release water is crucial for comprehending the resilience of native vegetation in the face of climate change. This knowledge can also enable the development of strategies to mitigate the effects of climate change and protect vital water resources.

## 1. Introduction

Bryophytes are an important part of the Azorean ecosystems, contributing significantly to the overall plant diversity of the archipelago (*n* = 480 species and subspecies) [1], even more than indigenous vascular plants (*n* = 209) [2,3]. The studied MOVECLIM transects of Terceira and Pico Islands, encompassing elevational gradients from the coast to the summit of the islands, also present high values of bryophyte diversity [4,5], much higher than the number of indigenous vascular plant species [4,6,7]. This high diversity is also related to different ecosystem services performed by bryophytes, namely those related to water retention and stormwater management.

Bryophyte occurrence (presence and abundance) is influenced by many factors, such as light-shade conditions [8], age and composition of the forest [9,10], factors related to the chemistry of the substrate (e.g., pH, nutrient status) [11,12], and clearly also by climatic variables such as temperature, precipitation, and moisture regime [13,14]. In the Azores, many of these factors combine to ensure an exceptional richness and abundance of bryophyte species. Indeed, the mild temperatures and high and stable values of relative humidity throughout the year [15] favor the presence of bryophytes in a large variety of substrata [16,17,18].

Water is essential to life and vital to all processes related to the good metabolic functioning of plants. Due to their simple morphological features, lack of roots, and absence of true vascular tissues in the sporophyte generation, bryophytes depend almost exclusively on external water supplies [19,20]. Thanks to their poikilohydric nature, mosses and liverworts photosynthesize and grow actively when conditions are propitious, that is, if liquid water or high relative humidity is available; otherwise, they dry out and stay metabolically inactive (review in Proctor and colleagues [21]). This efficient survival strategy allows bryophytes to colonize impermeable substrata (e.g., rocks and leaves), even in dry and harsh environments where higher plants are unable to survive [22].

However, the absence of water for longer periods, depending on the taxonomic group and species, will lead to the destruction of cells by plasmolysis and, eventually, to the death of the plant [23]. It is acknowledged that bryophytes’ structure and maturity change the capillarity features of the species and have functional consequences both in their potential water holding ability [24,25,26,27] and in their resistance to water loss [28]. For instance, the morphological organization of the shoots in colonies [29], leaf arrangement (e.g., succubous and incubous) [30], presence of leaf hair-points [31], thickness (e.g., costa, lamellae) and shape (e.g., lobules, water sacs) [28,32,33,34], presence of surface wax [32], cell thickenings, and cell specialization (e.g., hyaline cells) are some of the features that improve water holding capacity. Consequently, the architecture of bryophytes acts as an efficient strategy to achieve, in a balanced way, the water economy and light capture, as well as carbon and nutrient acquisition. These traits can be influenced by their environment and substrata [21,35,36]; for instance, species of the genus *Frullania* often grow as mats in exposed environments but as pendants in humid and sheltered forests in the Azores.

In plant physiology, a useful measurement to evaluate a plant’s water status is to determine the water content of the species, measured as the wet weight per dry weight [37]. The water content of the species in the field—field water content (FWC)—informs us about the amount of water that a species holds at a specific moment. Köhler and colleagues [38] determined the water content of epiphytic bryophytes in situ in an old-growth forest in Costa Rica, finding values ranging from 36% (dry periods) to 418% (wet periods) of dry weight, thus showing that bryophytes can significantly increase the overall water storage capacity of montane forest canopies. Another cryptogamic group, fruticose and foliose lichens, that share the capacity to rapidly absorb water, store between 150% and 350% of their dry weight [39,40], while Pypker and colleagues [41,42], studying epiphytes (foliose lichens, fruticose lichens, and bryophytes), found field water storage capacity values between 80% and 550%. The water storage capacity of two epiphytic liverworts (*Bazzania decrescens* and *Mastigophora diclados*) in a tropical montane cloud forest exceeded maximum values of 1000% of dry weight [43].

Relative water content (RWC) is another useful physiological measurement as it provides insights into both the water status and the health of plants. This indicator includes not only the field weight, but also the plant-saturated weight, informing us about the plant’s hydration condition relative to its maximum water holding capacity. This indicator shows the potential amount of water that may be measured at a particular site. Fully turgid crop plants reach almost saturation values (RWC = 98%), while initial wilting plants show intermediate values (RWC = 60–70%), and severely desiccated and drying leaves exhibit lower values (RWC = 30–40%) [44]. Notwithstanding, bryophytes are only considered desiccated, thus unable to support the metabolism, when holding water equivalent to about 5–10% of their dry weight (desiccation, 50% RH at 20 °C) [28,45,46]. The ability of bryophytes to withstand desiccation is exemplified by their low water content, which serves as a remarkable life strategy.

Despite the crucial role of bryophytes in ecosystems, particularly in water storage [42,46], and their ability to reflect rapid changes in water at the microhabitat scale [47], they have received relatively less attention compared to other plant groups. There is still a significant need for a better understanding and quantification of their water relations, especially in temperate regions such as the Azores, where they are abundantly present.

Thus, the purpose of this study is to investigate, for one year, the hydration level of some of the most common and representative bryophytes found on three native vegetation sites along an elevational gradient on Terceira Island (Azores), and consequently improve knowledge on their contribution to water availability in nature. The research questions addressed here are as follows: (i) How do the field water content and the relative water content of 14 bryophyte species vary throughout the year in three distinct native vegetation stands along an elevation transect (40 m, 683 m, and 1012 m above sea level (a.s.l.))? (ii) How do climatic variables such as temperature, precipitation, relative humidity, and vapor pressure deficit impact the field water content and relative water content values of these species across the year?

## 2. Results

### 2.1. Variations of Field Water Content in Bryophytes along an Elevation Gradient

The main objective of this study was to examine the relationship between field water content (FWC) of 14 different bryophyte species, representing a wide variety of taxonomic categories (2 divisions, 4 classes, 7 orders, and 12 families), within the Azorean native vegetation across one year. Six liverwort species (*Bazzania azorica*, *Herbertus azoricus*, *Lepidozia cupressina*, *Plagiochila bifaria*, *Scapania gracilis*, *Frullania acicularis*) and eight moss species (*Sphagnum subnitens*, *Polytrichum commune*, *Campylopus brevipilus*, *Campylopus shawii*, *Isothecium prolixum*, *Myurium hochstetteri*, *Thuidium tamariscinum*, and *Trichostomum brachydontium*). Due to species sharing among sites, there are FWC values for 19 species/locations. As expected, FWC values varied throughout the year for each species (Appendix A).

At the lowest elevation site, Farol da Serreta (40 m a.s.l.), the field water content (FWC) values ranged from 0.24 g/g to 4.78 g/g. Notably, there is a significant contrast between the FWC values observed during the summer months and those of the other seasons (Appendix A). The FWC values of the three species are generally within the same range; however, the moss *C. brevipilus* displayed the lowest FWC during the summer months of July and August, retaining however a hydration level consistently above 20%.

At mid-elevation, Pico da Lagoínha (683 m a.s.l.), moss species exhibited the widest range of FWC values, varying from 0.50 g/g (*Myurium hochstetteri*, September) to 21.25 g/g (*Sphagnum subnitens*, February) (Appendix A).

The sole representative of Order Sphagnales, *Sphagnum subnitens*, displayed the widest range of FWC values, ranging from 7.55 to 21.25 g/g (range: 13.70 g/g). From June to September, FWC values reached their lowest, significantly differing from values recorded in other months (*p* < 0.05). It is noteworthy that even these ‘lower’ values are considerably higher than most other FWC values from various species, as anticipated due to their unique plant architecture and areolation. While March exhibited the lowest FWC values among the three pleurocarpous mosses, the values were also relatively low during the warmer months of June to September. Remarkably, the large oceanic moss, *Myurium hochstetteri*, known for its concave leaves, displayed the highest FWC value among pleurocarpous mosses (13.34 g/g; April) but also the lowest FWC value (September), demonstrating an impressive range of variation.

The three foliose liverwort species found at this site also showed substantial variation in their field water content values, ranging from 0.52 g/g (*Frullania acicularis*, September) to 11.36 g/g (*Plagiochila bifaria*, May) (Appendix A). The only studied representative of Order Porellales (*F. acicularis*) tended to present the lowest FWC values during the year, while the two representatives of Order Jungermanniales showed higher values, especially from August to November.

Near the highest point of the island, Serra de Santa Bárbara (1012 m a.s.l.), FWC values ranged from 1.04 g/g to 20.35 g/g (Appendix A). The liverwort *Frullania acicularis* showed the lowest value in the summer (August), while the moss *Sphagnum subnitens* presented the highest one in the spring (April).

At this site, the median FWC values of the six foliose liverworts show certain similarities in water content throughout the year, with *Herbertus azoricus* and *Plagiochila bifaria* presenting the lowest effective water content and *Lepidozia cupressina* and *Bazzania azorica* exhibiting the highest FWC values. (Appendix A). The highest median FWC value is always found during winter and the lowest values during the summer and autumn months, although there are wide variations among replicates.

The FWC values of the three mosses studied on the highest elevation site are quite different (Appendix A). *Sphagnum subnitens*, exhibits a somewhat similar pattern to what happened in the mid-elevation site, showing the lowest FWC value in August (9.63 g/g) and the highest during April (20.35 g/g), encompassing a range of more than 10 g. This pattern is very different from what is found in the only representative of Polytrichaceae. The FWC range of *Polytrichum commune* is the lowest of all assessed species, varying from 2.21 g/g in October to 4.14 g/g in June, never showing expressive differences throughout the year. The large acrocarpous moss, *Campylopus shawii*, presented FWC values varying from 1.59 g/g in August to 8.75 g/g in October.

### 2.2. Relationship between Climate Variables and Field Water Content

The correlation of three key climate variables—precipitation, temperature, and relative humidity—was analyzed to understand the factors influencing FWC. These variables were selected due to their known association with water availability and potential impact on vegetation. Generalized linear models (GLM) explored the relationship between species’ field water content and three climatic variables across 14 species at three elevation levels (Table 1, Table 2 and Table 3).

At the lowest elevation site, Farol da Serreta (40 m a.s.l.), precipitation primarily influenced the field water content for all three species (Table 1). Additionally, the liverwort *Frullania acicularis* exhibited a significant positive response to relative humidity. The highly significant results of the omnibus models (*p* < 0.000) indicate that these associations are highly unlikely to have occurred by chance.

At mid-elevation, Pico da Lagoínha (683 m a.s.l.), relative humidity played a key role in determining the field water content for all seven species (Table 2). Furthermore, two liverwort species (*P. bifaria* and *F. acicularis*) and two mosses (*I. prolixum* and *M. hochstetteri*) showed significant positive interactions with precipitation. Once again, the highly significant results of the omnibus models (*p* < 0.000) support the notion that these relationships are not random.

At the highest elevation site, Serra de Santa Bárbara (1012 m a.s.l.), a significant model was not found for all species (Table 3). For seven out of nine species, temperature was the main driver of the field water content—all studied bryophytes except for the moss *Polytrichum commune*, which did not appear to respond to any climatic variable, and the liverwort *Lepidozia cupressina*, which responded solely to precipitation. Additionally, the liverwort *Bazzania azorica* and the moss *Campylopus shawii* reacted to both temperature and precipitation, while *F. acicularis* consistently retained relative humidity in the GLMs. The majority of omnibus models yielded highly significant results (mostly *p* < 0.000), indicating that chance is an unlikely explanation for these findings. However, it is worth noting that for *Plagiochila bifaria*, the omnibus model did not show significant differences when compared against the intercept-only model.

### 2.3. Field Water Content of the Species Shared between Different Elevations

Four plant species, including three liverwort species (*Frullania acicularis*, *Plagiochila bifaria*, and *Scapania gracilis*) and one moss species (*Sphagnum subnitens*), were monitored across multiple sites throughout the year due to their sufficiently large populations. *Frullania acicularis* was assessed across a wide elevational range, while the other three species were studied at mid and high elevations, specifically Pico da Lagoínha and Serra de Santa Bárbara (Appendix A; Table 4).

*Frullania acicularis* showed a gradual increase in its mean annual field water content (FWC) values along the elevational gradient, with values ranging from 1.40 g/g [FS] to 2.93 [PL] and 4.59 g/g [SB]. The lowest FWC values were consistently found in plants from Farol da Serreta at 40 m elevation, while the highest values were consistently found in plants from Serra de Santa Bárbara at 1012 m elevation. *Plagiochila bifaria*, commonly found at middle to high altitudes in the Azores, displayed its maximum FWC value at mid-elevation in May. At Serra de Santa Bárbara, the mean yearly average FWC was higher, with the maximum value recorded in January. *Scapania gracilis* consistently exhibited higher FWC values, both in terms of median and maximum, at the high-elevation site Serra de Santa Bárbara compared to Pico da Lagoínha.

As for the moss species, *Sphagnum subnitens*, the FWC values were quite similar between the two studied sites. However, during the summer months, the plants collected at Serra de Santa Bárbara displayed higher hydration levels compared to those collected at Pico da Lagoínha.

The generalized linear model explaining FWC values for *Frullania acicularis* included all three climate factors. *Plagiochila bifaria* and *Sphagnum subnitens* responded to both precipitation and temperature, while the key variables explaining the FWC values of *Scapania gracilis* were temperature and relative humidity. Highly significant results were obtained for all omnibus models.

### 2.4. Seasonal Variations in the Relative Water Content (RWC) of Bryophytes in Native Vegetation

Throughout one year, at three study sites, we calculated both field water content (FWC) and relative water content (RWC) values for each species (Appendix A and Appendix B). RWC represents the plant tissues’ water status or hydration level, shown as a percentage relative to the maximum water holding capacity of plant cells under fully hydrated conditions.

The lowest RWC’ values for the 19 taxa were mostly recorded in the warmer summer months: nine taxa in August and five taxa in September. On the other hand, the highest RWC values varied across different months, depending on the species, but never occurred during the summer (Appendix B). The minimum RWC value was recorded at Farol da Serreta (RWC = 1.49%) and the maximum at Serra de Santa Bárbara (RWC = 100%), a maximum reached by several liverworts, namely the endemic species *Bazzania azorica* and *Herbertus azoricus*, as well as *Scapania gracilis*. Interestingly, many species exhibited low RWC values in March, even at the highest elevation site.

At Farol da Serreta, the RWC values varied from 1.49% to 33.70%, and only one sample, in December, reached one-third of its maximum hydration capacity (*Frullania acicularis*; 33.7%). The lowest hydration status was observed in *Campylopus brevipilus* from June to August.

At the mid-elevation site, Pico da Lagoínha, more than one-tenth of all samples (*n* = 78) reached over 50% of their maximum hydration level (Appendix B). The RWC exhibited a wide range of values, spanning from 2.57% to 100%. The lowest RWC value was observed in a September sample of *Myurium hochstetteri*, while several samples of the liverwort *Plagiochila bifaria* and the moss *Isothecium prolixum* achieved the highest RWC values in May, during the spring season.

Meanwhile, at Serra de Santa Bárbara (1012 m), nearly half of all samples (*n* = 251) were adequately hydrated, with more than 50% RWC values. At the highest elevation site, RWC values ranged from 8.21% to 100% (Appendix B). Notably, during the winter season, four out of the six targeted liverwort species demonstrated average RWC values above two-thirds of their water-holding capacity while the only studied representative of the class Sphagnopsida, *Sphagnum subnitens*, exhibited RWC values consistently below 42% at both Pico da Lagoínha and Serra de Santa Bárbara. At the highest site, this moss showed RWC values ranging from 18.60% (August) to 39.32% (April). Despite having significant hydration potential, *Sphagnum subnitens* consistently displayed the lowest RWC values throughout the year.

In contrast, the large acrocarpous species, *Polytrichum commune*, a representative of the class Polytrichopsida, known for generally having lower hydration potential, consistently maintained relatively high hydration levels throughout the year. Even during the summer season, when other species tend to experience lower hydration levels, *Polytrichum commune* maintained a minimum RWC value of over 40% (RWC[October] = 45.40%). Its RWC values consistently remained above two-thirds of the maximum capacity throughout the seasons, with a notably high RWC value in June (RWC[June] = 81.90%). 

Another acrocarpous moss species studied in Serra de Santa Bárbara, *Campylopus shawii* (class Bryopsida), showed a similar pattern to the congeneric species, *Campylopus brevipilus*, with the lowest values observed during the summer season (July–August).

## 3. Discussion

In this study, it was investigated how 14-bryophyte species, common in native vegetation of Terceira Island (Azores) [48], with known absolute water contents [49], adapt to variations in water availability across three elevations throughout the course of one year. Additionally, it was also examined whether these species experienced a water deficit and what their hydration level was in relation to their maximum holding ability, thus exploring their contribution to the ecosystem’s water compartment. The 14 species encompass diverse taxonomic groups, showcasing a variety of morphological characteristics, life forms, substrates, and habitat preferences. Four species were studied across distinct elevation sites, adding further variation to their ecological attributes.

Apart from their role in nutrient cycling [50], erosion control [51], and biodiversity promotion [2,52,53], bryophytes particularly contribute to the ecosystem’s water compartment in a number of ways, including water retention and movement [42,54,55], prevention of water loss [38], water infiltration, and soil moisture [46,56]. However, knowledge regarding the contribution of different species at different elevations, particularly in rich temperate ecosystems, is practically nonexistent. In fact, many of the results regarding the water content of bryophytes cannot be directly compared to this study, as species were not individually examined but rather grouped into categories such as “epiphytes” [41,42,54,57,58] or “soil and logs” bryophyte’ communities [46], while other studies focused on different properties such as desiccation tolerance [29].

This section aims to provide a detailed analysis of the field water content (FWC) of several species and its relationship with various explanatory variables across three different elevation sites. Additionally, it will explore the implications of the relative water content (RWC) values found relative to the role played by bryophytes in mitigating the adverse impacts of stormwater. Regarding ecosystem services, FWC primarily reflects water retention capabilities, whereas RWC illustrates bryophytes’ role in preventing the detrimental consequences of extreme precipitation events.

### 3.1. How Did Elevation Impact the Bryophyte Field Water Content?

**Figure 1:**
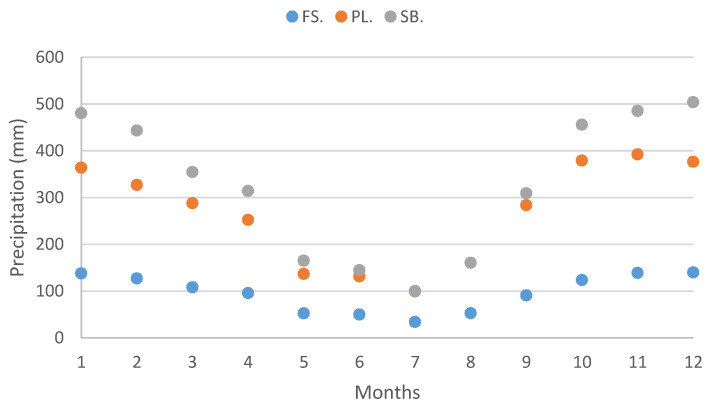
Mean precipitation values (mm) for each studied site (FS: Farol da Serreta; PL: Pico da Lagoínha; SB: Serra de Santa Bárbara) on Terceira Island (Azores) across the 12 months. Climate data were obtained from CLIMAAT.

**Figure 2:**
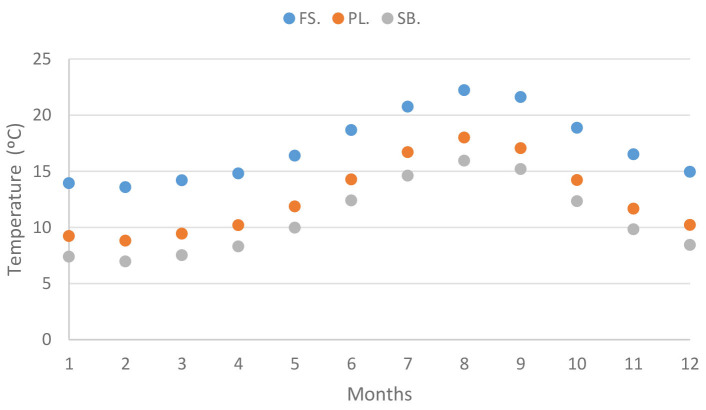
Mean temperature values (°C) for each studied site (FS: Farol da Serreta; PL: Pico da Lagoínha; SB: Serra de Santa Bárbara) on Terceira Island (Azores) across the 12 months. Climate data were obtained from CLIMAAT.

**Figure 3:**
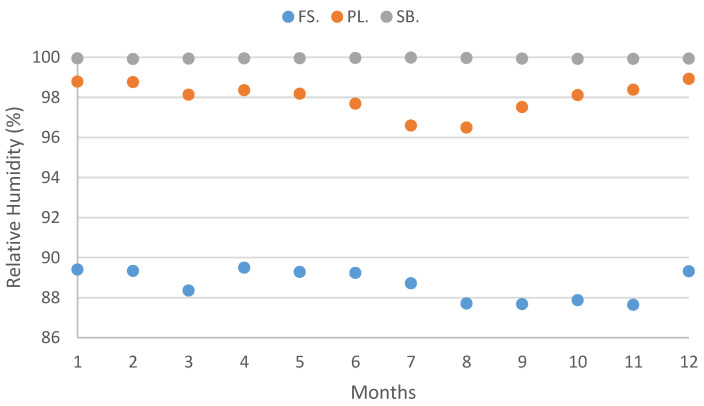
Mean relative humidity values (%) for each studied site (FS: Farol da Serreta; PL: Pico da Lagoínha; SB: Serra de Santa Bárbara) on Terceira Island (Azores) across the 12 months. Climate data were obtained from CLIMAAT.

**Figure 4:**
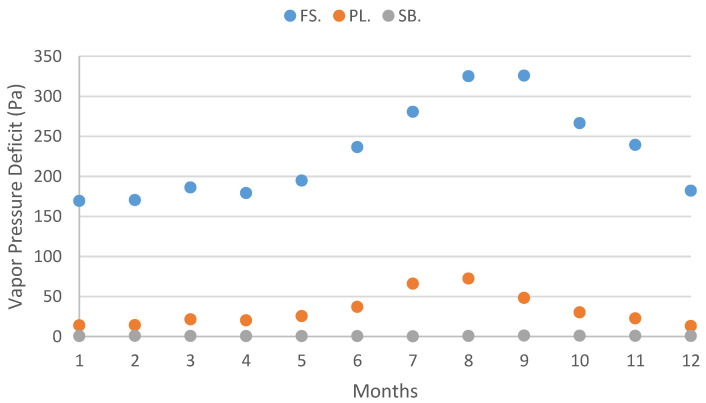
Mean vapor pressure deficit values (Pa) for each studied site (FS: Farol da Serreta; PL: Pico da Lagoínha; SB: Serra de Santa Bárbara) on Terceira Island (Azores) across the 12 months. Climate data was determined according to Monteith and Unsworth [84].

Elevation is widely recognized as a reliable and commonly used proxy for studying climate variables [6]. In the study, conducted on Terceira Island, specific research sites were intentionally selected at low (40 m a.s.l.), medium (683 m a.s.l.), and high (1012 m a.s.l.) elevations. The investigation encompassed an analysis of various climate variables, including precipitation, temperature, relative humidity, and water vapor pressure (Figure 1, Figure 2, Figure 3 and Figure 4), in relation to field water content and relative water content.

### 3.2. Lowland—Climate and Environmental Factors

Three species were monitored at the lowland site, Farol da Serreta, namely, *Frullania acicularis*, *Campylopus brevipilus*, and *Trichostomum brachydontium*. These species were collected from black volcanic rocks within a coastal *Erica azorica* scrub [4,59]. Given the challenging conditions of drought and salty winds resulting from their exposed location, the field water content (FWC) values of these species consistently remained low, with an annual average not exceeding 1.39 g of water per gram of dry weight (g/g).

Nevertheless, the hydration levels of the three species never dropped below 20% (not even in summer), which is twice the threshold value for complete desiccation in most bryophytes (10%) [28] and further exceeds the tolerance limit of certain desiccation-tolerant species such as *Syntrichia* (*Tortula*) *ruralis* and *Racomitrium lanuginosum* (5%) [60,61]. This indicates that the species under study had sufficient water available to support their metabolic processes, allowing for carbon gain and growth throughout the four seasons of the year.

In fact, even if this site has many characteristics of a dry habitat, its permanent high relative humidity (always above 80%) is probably a relevant factor helping species not to desiccate, but to grow and survive over time. Research on desiccation tolerance in *Syntrichia norvegica* has demonstrated that maintaining plants at 75% relative humidity conditions leads to less structural damage [62]. Indeed, values of that magnitude or higher are experienced in Farol da Serreta, even during the summer (Figure 3), despite lower precipitation values compared to the other two sites on Terceira Island (Figure 1). The moderate temperature values, remaining below 25 °C (Figure 2), likely contribute to preventing major cell damage and increasing survival rates [63].

At low elevation, bryophytes primarily rely on water from precipitation events, which directly influences the hydration levels of the three species. This finding aligns with the study by Bates [64], which showed that growth correlation with seasonal precipitation is most common in ectohydric species growing on hard substrata and bryophytes with limited plant-soil contact.

### 3.3. Lowland—Traits and Species/Taxa Comparisons

As drying rates can be very rapid in exposed habitats, species occupying such territories usually present xerophytic adaptations to reduce the available surface and control water loss, such as clump architecture [31,65], or other morphological features [66].

At Farol da Serreta, the three studied species exhibit distinctive morphological adaptations. For instance, *F. acicularis* displays helmet-shaped lobules; *C. brevipilus* has white hair points; and *T. brachydontium* exhibits twisted leaves on drying [49]. Interestingly, in the study conducted by Cruz de Carvalho and colleagues [29], the moss *C. pyriformis* showed a lower recovery rate after rapid drying, a feature attributed to its looser colony structure. The same feature may help to explain the consistent low values of FWC of the congeneric *C. brevipilus* found in this study. The moss species *Leptodontium exasperatum* [67], growing in an old-growth Costa Rican forest, displayed hydration values ranging from 24 ± 1% [SD] during the dry season to 406 ± 31% [SD] during the rainy season. These values align with the findings for the only Pottiacea in this study, *T. brachydontium*, which exhibited average hydration values of 38 ± 1% [SD] in summer and 166 ± 7% [SD] in winter months (cf. Appendix A).

### 3.4. Mid- and Highland Sites—Climate and Environmental Factors

The mid- and highland sites, Pico da Lagoínha and Serra de Santa Bárbara, share more similarities with each other than with the lowest elevation site. Those areas are characterized by native forest vegetation, dominated by the endemic gymnosperm *Juniperus brevifolia* [4,59], and exhibit a high richness and coverage of bryophytes, with a considerable number of species in common, including *Sphagnum* growing under the trees [48].

Such wet, deeply shaded, and intricate forest habitat [4,18,68] provides diverse substrates for bryophyte colonization, creating closely knit communities that enhance water retention [69,70]. These sites maintain near-saturation atmospheric conditions throughout most of the year, with relative humidity consistently above 95% and a small saturation deficit (Figure 3 and Figure 4). Consequently, bryophytes in these areas are not expected to experience water scarcity. In fact, the lowest hydration values observed in this study, exhibited by *Myurium hochstetteri* and *Frullania acicularis* at mid-elevation during the summer months, are approximately 50%, which is not considered stressful for bryophytes. According to Proctor [61], species inhabiting shady and wet habitats can tolerate water contents around 15–20% of dry weight when in equilibrium with an atmosphere with a relative humidity of approximately 60–80%. Both Pico da Lagoínha and Serra de Santa Bárbara sites provide shady and humid conditions with abundant tree cover, reducing the rate of water loss for bryophytes and enhancing their water retention capabilities.

At mid-elevation, Pico da Lagoínha, relative humidity positively and significantly influences the field water content (FWC) of the assemblage of bryophytes (Table 2). At this altitude, relative humidity is the primary predictor for FWC, and it is consistently included in all Generalized Linear Models (GLMs) for the seven species. At high elevation, Serra de Santa Bárbara (1012 m a.s.l.), the bryophyte assemblage shows a stronger negative influence of temperature in the FWC of seven out of the nine species. However, precipitation is still retained as a factor in three GLMs, while relative humidity remains a factor in one (Table 3).

Certain species demonstrate notably high average maximum hydration levels, particularly during the winter months: the leafy liverworts *Bazzania azorica* and *Lepidozia cupressina* reach hydration levels of approximately 1000%, while the moss *Sphagnum subnitens* exhibits an impressive hydration level of nearly 2000%. In fact, certain specimens of this species reached a field water content FWC_[max.]_ = 21.25 g/g (2125% hydration), indicating that they were carrying 21 times more water than their own dry weight, which is in line with their absolute water content values [49]. These findings support the idea that, besides *Sphagnum* species, liverworts and other mosses are also important to ecosystem water content [47,49,71,72,73]. In fact, at mid and low elevations, FWC values consistently exceeded the proposed threshold of “three times the dry weight” suggested by Frahm and Pócs [57].

### 3.5. Mid- and Highlands—Traits and Species/Taxa Comparisons

Morphological characteristics, such as cell arrangement and growth form, likely contribute to the exceptional water-holding capacity and/or evaporation reduction verified among these bryophyte species. Plants in sheltered and moist habitats often have longer, nerveless leaves and larger cells that help retain water effectively [74]. In fact, a transition from mosses with twisted and shorter leaves with nerves at lower elevations to mosses with longer, untwisted leaves and shorter or absent nerves at higher elevations has already been observed on Terceira Island [75]. A case in point can be found in the genus *Campylopus*: *C. brevipilus*, found at lower elevation, is characterized by short leaves, straight and erect when moist but becoming slightly reflexed when drying, while *C. shawii* displays longer leaves, with the lower ones spreading away from the stem, while the leaves near the top of the shoot are either erect or curved when moist, becoming only slightly wavy on drying [49].

The liverworts *Bazzania azorica* and *Lepidozia cupressina* tend to grow in wefts, with many or fewer ramifications, presenting incubous leaves and underleaves, while *Scapania gracilis* presents lobed leaves that have slower rates of evaporation than undivided leaves of similar surface area [30,71], and *Plagiochila bifaria* and *Polytrichum commune*, with their decurrent bases, facilitate water movement. The oceanic moss *Myurium hochstetteri* has very concave leaves, and *Thuidium tamariscinum* presents cell ornamentations, which favor the collection and retention of water since spaces between papillae form a capillary conducting system and alter the boundary layer, reducing water loss [33,71]. The genus *Sphagnum* includes a typical areolation pattern, with leaves featuring large hyalocysts that allow for efficient water storage and easy flow; moreover, the distinctive growth form of this genus, characterized by pendent and patent branches, facilitates water retention through capillary action across various axes [49,76].

On the other hand, *Polytrichum commune* presents the lowest average FWC values in Serra de Santa Bárbara (Appendix A). It is well known that Polytrichaceae species have some cuticular resistance to water loss, namely water-repellent surfaces, so cell saturation is not reached as quickly and easily as in other bryophytes [61,77]. Besides, as one of the most endohydric bryophytes analyzed in this study [49], this species tends to be restricted to places with a continuous water supply. In this study, *P. commune* samples behaved as an endohydric species, with their level of hydration and metabolic condition likely determined by the water potential of the underlying substrate [56], namely *Sphagnum* sp. turfs, and only to a lesser extent by the monthly inputs of precipitation, a factor that also did not predict the growth of two thalloid liverworts (*Pallavicinia lyelli* and *Pellia epiphylla*) in Southern England [64], also approaching endohydric strategies.

The response of bryophytes’ FWC to climate variables was, as expected, positive with precipitation and relative humidity, and negative with temperature and vapor pressure deficit. It is important to emphasize that the climate data utilized in the study does not directly reflect the precise climatic conditions during the collection periods. Instead, it comprises macro-scale climatic series [15,78]. However, despite this limitation, the data still offer valuable explanatory insights in the derived models. It should be acknowledged that other factors such as micro-climate, environmental variations, and specific local climate conditions may also contribute to the functional water capacity of the species.

Future climate changes, including temperature increases and altered precipitation patterns [79], may induce drought events that diminish water availability and impact bryophytes’ field water content (FWC). Ferreira and colleagues [80] used ensemble forecasting to assess the distribution of 19 Macaronesian endemic species and projected that 79% of these bryophytes would experience climate space loss, predominantly in coastal areas already experiencing heightened land use changes. Currently, bryophytes at the study sites maintain year-round hydration, but differing climatic scenarios could disrupt their hydration levels, especially at lower elevations. Climate warming poses a substantial threat to bryophytes’ water storage capacity [46,79,80]. Thus, it is crucial to comprehend and anticipate the potential consequences of climate change for the survival of both these indicator organisms [81], and the human species.

### 3.6. Did Bryophytes Reach Their Maximum Water Retention Capacity?

The relative water content (RWC) of the 14 selected species varies among the seasons (Appendix B). All bryophyte species were hydrated above the wilting point during the year [26]. The six species of the class Jungermanniopsida consistently had the highest relative water content (RWC) values, approaching their maximum capacity (RWC_max_ > 90%), throughout the seasons, except during the summer months. These leafy liverworts, which grow primarily on trees, experience greater exposure to climate conditions and have limited contact with soil water. Consequently, they exhibit the greatest fluctuation in RWC values compared to other species. Among the six mosses from the class Bryopsida, RWC tends to respond similarly to RWC of the studied liverwort species, presenting also high variation in their RWC values. A comparable study [26] showed that the RWC of several species over one year and the values obtained for the moss *Thuidium tamariscinum* were in accordance with this experiment (r_s_ = 0.79). Curiously, the minimum and maximum hydration levels of *Thuidium tamariscinum* were also recorded during the autumn months.

Contrariwise, the only representative from class Sphagnopsida, *Sphagnum subnitens*, was, on average, the least hydrated species in the field throughout the 12 months, showing an annual average of less than one third of its maximum potential (RWC_[Ss.]_ = 29.06%). These plants only reached 41% of their quite considerable maximum holding water ability [49]. In fact, this important capacity not only results in water accumulation during the summer but also proves especially valuable in mitigating the mechanical impacts of intense precipitation events during the winter, as emphasized in Azevedo’s study on Flores Island (Azores) [82].

A completely different strategy is exhibited by *Polytrichum commune* (class Polytrichopsida), which presents the lowest variation of its RWC values, showing almost constant average values of hydration throughout the year (RWC_[Pc.]_ = 57.65 ± 7.67% [SD]). As a preferentially endohydric species [49], *P. commune* would probably not be able to resist large fluctuations of water content. In effect, this species was collected interspersed with *Sphagnum* spp., so it is assumed that water was always available to it, as *P. commune* is efficient at withdrawing water from a substrate [71].

Bryophytes possess a high water storage capacity, making them valuable for mitigating the impacts of extreme precipitation events. This study demonstrates that across an elevation gradient on Terceira Island (Azores), most bryophyte species maintain suboptimal water content, indicating an important ecological role in preventing the effects of extreme precipitation events. Specific bryophyte species, like *Racomitrium canescens*, are increasingly being utilized for efficient stormwater management in urban areas [83].

## 4. Materials and Methods

### 4.1. Study Sites

#### 4.1.1. General Information

This study was conducted on Terceira Island, Azores archipelago, Portugal (38°40′ N; 27°20′ W), in the North Atlantic Ocean, between the European and North American continents [85].

Three native vegetation stands were selected at three elevations, therefore encompassing different temperature and precipitation regimes as well as different plant communities. The three collection sites are part of the Natural Park of Terceira Island: Farol da Serreta (FS, 40 m a.s.l.); Pico da Lagoínha (PL, 683 m a.s.l.)*;* and Serra de Santa Bárbara (SSB, 1012 m a.s.l.), near the highest point of the island. Except for the lowest site, the other two locations have a diverse and high cover of bryophytes. The lowland site is poorer in bryophyte cover, while Pico da Lagoinha and Serra de Santa Bárbara have similar bryophyte cover (Table 5). A spatial representation as well as a more detailed characterization of each site and a thorough description of the vegetation may be found in Henriques and colleagues [4].

#### 4.1.2. Climate Data

Three climatic variables, precipitation (P; mm), temperature (T; °C), and relative humidity (RH; %) were obtained from the CIELO Model [78]. This physical model estimates the monthly values for the Azores archipelago, based on the previous climatic series of 30 years. Thus, these data do not correspond to the climate data at collection time, which may lead to some inconsistencies. While using climate data from generalist sites like Windguru (https://www.windguru.cz/; accessed on 15 July 2014) could have been an alternative, these sources provide one data point for the entire island, often obtained from an urban area, with very different climate conditions from the ones tested. Hence, the values estimated by the CIELO climate model offer more precise and site-specific data for our study.

The vapor pressure deficit (VPD) was calculated according to Monteith and Unsworth [84]; this compound variable includes information about temperature and relative humidity and is quite useful in plant physiology and ecology studies, since it informs us about the drying power of the air, which consequently affects the relative rates of growth and transpiration of plants [84]. The equations are as follows:

(1) $$VPD=\frac{100-RH\;}{100}\times SVP$$

(2) $$SVP\;\left(Pascals\right)=610.7\times 10^{7.5T\left(237.3+T\right)}$$

in which *RH* is the relative humidity, *SVP* is the saturated vapor pressure, and *T* is the temperature.

Climate data was organized per site and month (Figure 1, Figure 2, Figure 3 and Figure 4) (Appendix C).

Precipitation increases with elevation, and at each site, it is higher in the autumn and winter months (Figure 1; Appendix C). The precipitation amplitude ranged from a minimum of 34.20 mm observed at the lowest elevation site, Farol da Serreta, in July and a maximum of 504.00 mm at the top of the island, Serra de Santa Bárbara, in December.

Values of temperature are negatively correlated with elevation; the lowest ones were recorded near the highest point of the island and the highest ones at sea level (Figure 2; Appendix C). Average temperature values ranged from a minimum of 7 °C at Serra de Santa Bárbara, in February, to a maximum of 22 °C at Farol da Serreta, in August.

Relative humidity showed extremely high values, all above 87%, even at the lowest elevation site, Farol da Serreta, while values showing almost complete saturation were found at the highest elevation site, Serra de Santa Bárbara (Figure 3; Appendix C). These data indicate that air currents do not appear to be diminishing water availability at the highest elevation site under consideration.

The vapor pressure deficit values varied considerably among the three sites. They were higher in the lowland site (Farol da Serreta: 169.39–325.88 Pa), indicating a high level of air dryness, and lower in the highland site (Serra de Santa Bárbara: 0.34–1.19 Pa), where the air is constantly humid (Figure 4; Appendix C).

It is clear from the above that in the Azores, there are two main periods concerning precipitation and temperature. In fact, the median values of precipitation are similar throughout the autumn and winter months and much lower during the spring and summer, while the lowest median temperature values are found during the winter and spring and the highest during the autumn and summer (Appendix C). 

On the other hand, the values of relative humidity are extremely high over the four seasons and the three sites, even at 40 m a.s.l., while Vapor Pressure Deficit discriminates the lowest site (Farol da Serreta) against the other two (Pico da Lagoínha and Serra de Santa Bárbara), where VPA’ values are considerably lower and much more stable along the year.

### 4.2. Study Species

A selection of 14 bryophyte species, representative of each studied site, was made according to three criteria:

(i) Species that were abundant and widespread enough on each studied site to withstand monthly collections for one year without significantly depleting the populations. At lower altitudes the selected bryophyte species are commonly found around the island, while the other species are typically present in mature native forests, where, even the endemic and conservation-concern species are capable of forming extensive and healthy patches [16,68,86];
(ii) Species that were large and relatively easy to recognize in the field [86];
(iii) Species that reflected taxonomic [1] and morphological diversity, including different life forms [55,87].

As a result of the diversity and abundance of bryophytes in the studied sites, three species were selected for the Farol da Serreta site, seven species for the mid elevational site of Pico da Lagoínha, and nine species for the highest site in Serra de Santa Bárbara (Table 6).

The liverwort *Frullania acicularis* was collected from the three sites, while *Plagiochila bifaria*, *Scapania gracilis*, and the moss *Sphagnum subnitens* were sampled from the two higher elevation sites, Pico da Lagoínha and Serra de Santa Bárbara.

### 4.3. Sampling Procedure in the Field

Firstly, in August 2014, all bryophyte populations of the selected species, were inspected and marked in the field. From September 2014 to August 2015, five replicates of each species were collected monthly from the same area under similar environmental conditions, namely, the same substrate, exposure, and height from the soil. Sample replicates were collected to sealed polyethylene vials, previously marked and weighed.

The collected area of each sample varied according to the size of the shoots. The smallest species (e.g., *Frullania acicularis*, *Trichostomum brachydontium*) were collected within an area of ca. 2.5 cm × 2.5 cm, while samples of medium-large species (e.g., *Myurium hochstetteri*, *Sphagnum subnitens*) occupied an area of ca. 5.0 cm × 5.0 cm.

### 4.4. Processing Samples in the Laboratory

The identification of all the bryophyte specimens was confirmed in the laboratory by M.C.M.C. and R.G. with the aid of a stereo microscope (Leica Mz12.5), a light microscope (Leica DM750), both from Leica Microsystems, Wetzlar, Germany, and different flora and identification keys [88,89,90,91,92,93]. Nomenclature follows the latest Azorean checklist [1], with taxonomic updates [86,94,95].

In the laboratory, fresh green shoots were first cleaned from debris and associated species, preserving the structure of the bryophyte colony. Around five hours after harvest, each sample was weighed in an A-150 balance (COBOS precision, Barcelona, Spain) to obtain the field weight. Immediately after that, samples were immersed for 12 h to achieve full turgor, then left to drip excess water over a wire rack until plants dripped less than one drop per minute, according to the protocol proposed by Coelho and colleagues [49]. This value accounts for all the water retained in the external capillarity spaces, as bryophytes can do in their colonies. Further, samples were oven-dried (48 h at 100 °C) and re-weighed to obtain the value of dry weight.

### 4.5. Data Analysis

#### 4.5.1. Water Content

Water Content was assessed as field water content (FWC; g/g—grams of water per gram of dry weight) and relative water content (RWC; %). The first measurement, FWC, is useful to understand the amount of water present in each species at a given time (collection time). The second measurement, RWC, informs us about the hydration level of the species related to their maximum holding water ability.

According to Watkins and colleagues [96], water content was obtained using the following relations:

(3) $$FWC=\frac{Mf-Md}{Md}\;$$

(4) $$RWC=\frac{\left(Mf-Md\right)}{\left(Ms-Md\right)}\times 100$$

in which *M_f_* is the fresh weight, *M_d_* is the dry weight after drying the plants in the oven (100 °C for 48 h), and *M_s_* is the saturated weight.

#### 4.5.2. Statistical Analysis

Since assumptions of normality were not satisfied neither for FWC nor for RWC values, even if using a logarithmic transformation of the data, the differences within and between populations for climate variables could not be tested using parametric tests. The relationships between the bryophytes’ monthly field water content (FWC) and climate variables were thus explored using generalized linear models (GLMs) with a gamma distribution and log link function as the response scale. Prior to analysis, auto-correlation higher than 75% was examined and addressed accordingly. The explanatory variables considered for each species and site included average seasonal precipitation (mm), average seasonal temperature (°C), and average seasonal relative humidity (%) [97]. The average seasonal vapor pressure deficit (Pa) was excluded due to its high correlation with relative humidity (>98%). To assess the overall significance of the predictor variables in the model, omnibus tests were conducted using likelihood ratio chi-square tests (LRC). A significant result, denoted by *p*-values below the chosen significance level (0.05), indicates that the full model is a better fit for the data than the mere intercept model, suggesting that at least one of the predictor variables is significantly related to the response variable.

All statistical analysis, including generalized linear models, was performed using the SPSS software (version 29; IBM SPSS Statistics, Chicago, IL, USA).

## 5. Conclusions

Bryophytes play a crucial role in providing valuable ecosystem services related to water retention, helping to prevent water loss from natural environments, and mitigating the impact of extreme climate events associated with precipitation. In the Azores, we investigated the water content of six liverwort species and eight moss species, which are commonly found in native vegetation stands of the region. To assess bryophytes’ water status, we utilized two water indicator variables: field water content (FWC), indicating the amount of water held by each species in the field, and relative water content (RWC), which represents the hydration level of a species relative to its maximum water holding capacity at any given moment.

An altitudinal trend was clearly detected in FWC, since moving from the lowest to the highest elevation sites (Farol da Serreta to Serra de Santa Bárbara), the main drivers of FWC changed. Precipitation plays a crucial role at the lowest elevation, relative humidity dominates at mid-elevation, and temperature becomes the primary driver at the highest elevation. Indeed, conducting studies along elevation gradients provides a convenient and efficient method for gathering data on species adaptation to climatic conditions, both at local and global scales.

It was also very advantageous to use a relatively high number of taxa, which allowed us to observe species-specific responses and exceptions. Different species exhibited varying responses to climatic variables, and some species exhibited unique trends that deviated from the general patterns observed across the elevation gradient. For example, the liverwort *Frullania acicularis* consistently responded to relative humidity at all three elevations. On the other hand, the liverwort *Lepidozia cupressina* only responded to precipitation, and the moss *Polytrichum commune* did not appear to respond significantly to any of the studied climatic variables. Furthermore, some species demonstrate significant positive interactions with multiple climatic variables. This suggests that the combined effects of different factors should be explicitly considered.

The hydration level of the studied bryophytes depends partially on climate conditions, benefiting from colder and wetter seasons (mostly during the winter and spring months) over warmer seasons (autumn and summer). Nevertheless, all bryophyte species showed that they were sufficiently hydrated throughout the whole year, actively contributing to the ecosystem’s water compartment and showcasing their high adaptability and potential for ecological and evolutionary adaptation.

From an ecological point of view, bryophytes are an important part of Azorean vegetation, since they occupy most of all available substrata—including rock, soil, tree bark, dead wood, humus, and even leaves—contributing to water fluxes and the maintenance of air humidity. Medium-high RWC values, even in the wettest seasons, highlight the important role that bryophytes play in ecosystems regarding water storage and safeguarding against extreme precipitation events.

Future climate changes have the potential to cause drought events, resulting in decreased water availability that can significantly impact the field water content (FWC) and relative water content (RWC) of bryophytes. Presently, most of the Azorean native vegetation areas, even at lowland, have a high atmospheric relative humidity, which protects the plants from drying out beyond recovery. However, under different climatic conditions, a shift towards a more drought-tolerant flora may occur, implying that the distribution and even the survival of some of the endemic species and/or oceanic-distributed species may considerably decrease.

Bryophytes play a critical role in reducing water loss from ecosystems, ensuring their own survival, and benefiting other organisms. Through water retention and subsequent evaporation, bryophytes contribute to maintaining air humidity, preventing excessive desiccation, and moderating microsite temperatures. This ecosystem service enhances water availability in Azorean ecosystems, particularly in forests, but is as important at lower elevations, where bryophyte colonies are establishing on bare rock surfaces, as pioneer species.

This study provides essential baseline data to better understand the role of bryophyte species in the water cycle within Azorean native vegetation and offers useful information for conservation, management, and sustainable use of ecosystem resources in Azorean vegetation. Likewise, these findings may be extrapolated to other temperate conditions, enhancing our understanding of ecosystem services and water management strategies.

## Figures and Tables

**Table 1:** Generalized linear model (GLM) analysis for the field water content (FWC) of the species at ‘Farol da Serreta’ (40 m a.s.l.), including omnibus tests. FWC data were collected on a monthly basis from September 2014 to August 2015, with five replicates per species per month; climate data were retrieved from CLIMAAT. Significant variables are indicated in bold.

Plant Species	Type III				Omnibus Test ^a^		
	Indicator Variable	Wald Chi-Square	df	Sig.	Likelihood Ratio Chi-Square	df	Sig.
*Frullania acicularis*	(Intercept)	4.41	1	0.036	30.44	3	<0.000
	**Precipitation**	6.13	1	0.013			
	Temperature	0.03	1	0.871			
	**Relative Humidity**	4.91	1	0.027			
*Campylopus brevipilus*	(Intercept)	2.08	1	0.149	32.98	3	<0.000
	**Precipitation**	10.09	1	0.001			
	Temperature	0.15	1	0.702			
	Relative Humidity	2.32	1	0.128			
*Trichostomum brachydontium*	(Intercept)	3.21	1	0.073	37.29	3	<0.000
	**Precipitation**	12.40	1	<0.000			
	Temperature	0.05	1	0.828			
	Relative Humidity	3.45	1	0.063			

**^a^**—Compares the fitted model against the intercept-only model.

**Table 2:** Generalized linear model (GLM) analysis for the field water content (FWC) of the species at ‘Pico da Lagoínha’ (683 m a.s.l.), including omnibus tests. FWC data were collected on a monthly basis from September 2014 to August 2015, with five replicates per species per month; climate data were retrieved from CLIMAAT. Significant variables are indicated in bold.

Plant Species	Type III				Omnibus Test ^a^		
	Indicator Variable	Wald Chi-Square	df	Sig.	Likelihood Ratio Chi-Square	df	Sig.
*Plagiochila bifaria*	(Intercept)	17.47	1	0.000	33.26	3	<0.000
	**Precipitation**	16.07	1	0.000			
	Temperature	2.98	1	0.084			
	**Relative Humidity**	18.46	1	0.000			
*Scapania gracilis*	(Intercept)	3.93	1	0.047	23.45	3	<0.000
	Precipitation	1.11	1	0.293			
	Temperature	0.02	1	0.876			
	**Relative Humidity**	4.32	1	0.038			
*Frullania acicularis*	(Intercept)	5.04	1	0.025	18.50	3	<0.000
	**Precipitation**	8.62	1	0.003			
	Temperature	0.37	1	0.543			
	**Relative Humidity**	5.36	1	0.021			
*Sphagnum subnitens*	(Intercept)	3.54	1	0.060	56.35	3	<0.000
	Precipitation	0.26	1	0.609			
	Temperature	1.45	1	0.229			
	**Relative Humidity**	5.03	1	0.025			
*Isothecium prolixum*	(Intercept)	11.27	1	0.001	32.27	3	<0.000
	**Precipitation**	12.44	1	0.000			
	Temperature	0.93	1	0.335			
	**Relative Humidity**	11.93	1	0.001			
*Myurium hochstetteri*	(Intercept)	7.92	1	0.005	41.50	3	<0.000
	**Precipitation**	5.13	1	0.023			
	Temperature	0.02	1	0.901			
	**Relative Humidity**	8.50	1	0.004			
*Thuidium tamariscinum*	(Intercept)	3.68	1	0.055	34.22	3	<0.000
	Precipitation	0.51	1	0.476			
	Temperature	0.08	1	0.777			
	**Relative Humidity**	3.96	1	0.047			

**^a^**—Compares the fitted model against the intercept-only model.

**Table 3:** Generalized linear model (GLM) analysis for the field water content (FWC) of the species at ‘Serra de Santa Bárbara’ (1012 m a.s.l.), including omnibus tests. FWC data were collected on a monthly basis from September 2014 to August 2015, with five replicates per species per month; climate data were retrieved from CLIMAAT. Significant variables are indicated in bold.

Plant Species	Type III				Omnibus Test ^a^		
	Indicator Variable	Wald Chi-Square	df	Sig.	Likelihood Ratio Chi-Square	df	Sig.
*Bazzania azorica*	(Intercept)	1.28	1	0.258	58.89	3	<0.000
	**Precipitation**	4.38	1	0.036			
	**Temperature**	18.29	1	0.000			
	Relative Humidity	1.26	1	0.261			
*Herbertus azoricus*	(Intercept)	0.00	1	0.987	17.73	3	0.001
	Precipitation	0.28	1	0.593			
	**Temperature**	10.14	1	0.001			
	Relative Humidity	0.00	1	0.990			
*Lepidozia cupressina*	(Intercept)	1.61	1	0.205	32.54	3	<0.000
	**Precipitation**	11.30	1	0.001			
	Temperature	3.25	1	0.071			
	Relative Humidity	1.62	1	0.203			
*Plagiochila bifaria*	(Intercept)	3.17	1	0.075	6.42	3	0.093
	Precipitation	0.83	1	0.362			
	**Temperature**	4.77	1	0.029			
	Relative Humidity	3.18	1	0.074			
*Scapania gracilis*	(Intercept)	2.73	1	0.098	14.03	3	0.003
	Precipitation	2.52	1	0.113			
	**Temperature**	8.09	1	0.004			
	Relative Humidity	2.75	1	0.097			
*Frullania acicularis*	(Intercept)	4.24	1	0.039	20.63	3	<0.000
	Precipitation	0.13	1	0.715			
	**Temperature**	21.61	1	0.000			
	**Relative Humidity**	4.27	1	0.039			
*Sphagnum subnitens*	(Intercept)	0.13	1	0.719	28.44	3	<0.000
	Precipitation	1.81	1	0.179			
	**Temperature**	12.68	1	0.000			
	Relative Humidity	0.14	1	0.708			
*Polytrichum commune*	(Intercept)	0.43	1	0.510	2.70	3	0.441
	Precipitation	0.00	1	0.978			
	Temperature	2.31	1	0.128			
	Relative Humidity	0.44	1	0.505			
*Campylopus shawii*	(Intercept)	2.80	1	0.094	29.37	3	<0.000
	**Precipitation**	11.15	1	0.001			
	**Temperature**	4.06	1	0.044			
	Relative Humidity	2.82	1	0.093			

**^a^**—Compares the fitted model against the intercept-only model.

**Table 4:** Generalized linear model (GLM) analysis for the field water content (FWC) of the species shared at different elevation’ sites, including omnibus tests. FWC data were collected on a monthly basis from September 2014 to August 2015, with five replicates per species per site per month; climate data were retrieved from CLIMAAT. Significant variables are indicated in bold. FS, Farol da Serreta; PL, Pico da Lagoínhs; SB, Serra de Santa Bárbara.

Plant Species (Sites)	Type III				Omnibus Test ^a^		
	Indicator Variable	Wald Chi-Square	df	Sig.	Likelihood Ratio Chi-Square	df	Sig.
*Frullania acicularis* (FS + PL + SB)	(Intercept)	0.560	1	0.454	108.886	3	<0.000
	**Precipitation**	7.747	1	0.005			
	**Temperature**	49.484	1	0.000			
	**Relative Humidity**	9.467	1	0.002			
*Plagiochila bifaria* (PL + SB)	(Intercept)	1.242	1	0.265	25.899	3	<0.000
	**Precipitation**	4.806	1	0.028			
	**Temperature**	13.758	1	0.000			
	Relative Humidity	2.718	1	0.099			
*Scapania gracilis* (PL + SB)	(Intercept)	9.650	1	0.002	50.903	3	<0.000
	Precipitation	0.086	1	0.770			
	**Temperature**	11.664	1	0.001			
	**Relative Humidity**	13.632	1	0.000			
*Sphagnum subnitens* (PL + SB)	(Intercept)	1.741	1	0.187	71.776	3	<0.000
	**Precipitation**	4.406	1	0.036			
	**Temperature**	36.920	1	0.000			
	Relative Humidity	0.181	1	0.670			

**^a^**—Compares the fitted model against the intercept-only model.

**Table 5:** Studied sites on Terceira Island (Azores) and their main characteristics (coordinates, altitude, exposure, and slope). Vascular vegetation type according to Elias et al. (2016) [59] (na—not applicable).

		Farol da Serreta (FS)	Pico da Lagoínha (PL)	Serra Santa Bárbara (SSB)
Geographic Variables				
Coordinates (N)/(W)		38°45′58.9″/27°22′32.4″	38°45′09.4″/27°19′53.7″	38°43′50.0″/27°19′18.7″
Altitude (a.s.l.)		40 m	683 m	1012 m
Exposure		270° W	150° S	20° N
Slope (°)		15.5	13.2	5.1
Vascular Vegetation				
Type		*Erica-Morella* coastal woodland	*Laurus* submontane forest	*Juniperus* montane woodland
Maximum height (cm)		300	800	160
Dominant phorophyte		*Erica azorica*	*Laurus azorica*	*Juniperus brevifolia*
Cover (%)		75	40	85
Bryophytes				
Richness (S)		22	56	50
Cover (%)	soil	10	95	90
	rocks	20	na	na
	epiphytes	5	70	90
Dominant species		*Frullania acicularis*	*Myurium hochstetteri*	*Scapania gracilis*

**Table 6:** Studied species identified with authorities and grouped by division (D), class and species abbreviations (Abb.), and life forms (LF), organized taxonomically. Each species was also identified by the type of substrate on which it was collected. Total sampling consists of five replicates per species, per site, and per month for each studied site (FS: Farol da Serreta; PL: Pico da Lagoínha; SB: Serra de Santa Bárbara) on Terceira Island (Azores). (db, divergent branches).

Sites	Division	Class	Species	Abb.	LF	Substrate
FS	Marchantiophyta	Jungermanniopsida	*Frullania acicularis* Hentschel and von Konrat	Fa	mat	rock
	Bryophyta	Bryopsida	*Campylopus brevipilus* Bruch et Schimp.	Cb	cushion	rock
			*Trichostomum brachydontium* Bruch	Tb	turf	rock
PL	Marchantiophyta	Jungermanniopsida	*Plagiochila bifaria* (Sw.) Lindenb.	Pb	turf	tree
			*Scapania gracilis* Lindb.	Sg	weft	tree
			*Frullania acicularis* Hentschel and von Konrat	Fa	mat	tree
	Bryophyta	Sphagnopsida	*Sphagnum subnitens* Russow et Warnst.	Ss	tall turf-db	humus
		Bryopsida	*Isothecium prolixum* (Mitt.) Stech, Sim-Sim, Tangney et D.Quandt	Ip	weft	tree
			*Myurium hochstetteri* (Schimp.) Kindb.	Mh	mat	tree
			*Thuidium tamariscinum* (Hedw.) Schimp.	Tt	weft	soil
SB	Marchantiophyta	Jungermanniopsida	*Bazzania azorica*	Ba	weft	tree
			*Herbertus azoricus* (Steph.) P.W.Richards	Ha	turf	tree
			*Lepidozia cupressina* (Sw.) Lindenb.	Lc	weft	tree
			*Plagiochila bifaria* (Sw.) Lindenb.	Pb	turf	tree
			*Scapania gracilis* Lindb.	Sg	weft	tree
			*Frullania acicularis* Hentschel and von Konrat	Fa	mat	tree
	Bryophyta	Sphagnopsida	*Sphagnum subnitens* Russow et Warnst.	Ss	tall turf-db	humus
		Polytrichopsida	*Polytrichum commune* Hedw.	Pc	tall turf	humus
		Bryopsida	*Campylopus shawii* Wilson	Cs	tall turf	soil

## Data Availability

The data presented in this study are available on request from the corresponding author.

## Appendix A

**Table A1:** Mean and standard deviation of the field water content (FWC; g/g) for all species observed at three study sites (FS: Farol da Serreta; PL: Pico da Lagoínha; SB: Serra de Santa Bárbara) on Terceira Island, Azores. The data span one year, with measurements taken monthly. Each data point is based on five replicates per species per month. Average values of liverworts (L) and mosses (M).

FWC	01_Jan	02_Feb	03_Mar	04_Apr	05_May	06_Jun	07_Jul	08_Aug	09_Sep	10_Oct	11_Nov	12_Dec
Farol da Serreta (FS)												
*Frullania acicularis*	0.83 ± 0.1	2.51 ± 0.2	0.41 ± 0.0	2.19 ± 0.3	2.77 ± 0.4	0.42 ± 0.0	0.37 ± 0.0	0.47 ± 0.1	0.43 ± 0.1	2.19 ± 0.3	0.98 ± 0.1	3.22 ± 0.6
*FS—Livertorts*	0.83 ± 0.1	2.51 ± 0.2	0.41 ± 0.0	2.19 ± 0.3	2.77 ± 0.4	0.42 ± 0.0	0.37 ± 0.0	0.47 ± 0.1	0.43 ± 0.1	2.19 ± 0.3	0.98 ± 0.1	3.22 ± 0.6
*Campy. brevipilus*	1.44 ± 0.5	2.96 ± 0.5	0.36 ± 0.1	3.50 ± 0.8	2.55 ± 0.3	0.31 ± 0.0	0.32 ± 0.1	0.26 ± 0.0	0.75 ± 0.2	3.14 ± 0.4	1.77 ± 0.3	3.10 ± 0.4
*Trich. brachydontium*	0.75 ± 0.1	2.12 ± 0.3	0.47 ± 0.0	1.37 ± 0.4	1.51 ± 0.2	0.38 ± 0.0	0.42 ± 0.1	0.36 ± 0.0	0.62 ± 0.3	1.55 ± 0.3	1.18 ± 0.2	2.12 ± 0.4
*FS—Mosses*	1.10 ± 0.5	2.54 ± 0.6	0.42 ± 0.1	2.44 ± 1.3	2.03 ± 0.6	0.34 ± 0.0	0.37 ± 0.1	0.31 ± 0.1	0.69 ± 0.2	2.35 ± 0.9	1.47 ± 0.4	2.61 ± 0.6
FS Total	1.01 ± 0.4	2.53 ± 0.5	0.41 ± 0.1	2.35 ± 1.0	2.28 ± 0.6	0.37 ± 0.1	0.37 ± 0.1	0.36 ± 0.1	0.60 ± 0.2	2.29 ± 0.7	1.31 ± 0.4	2.81 ± 0.7
Pico da Lagoínha (PL)												
*Plagiochila bifaria*	2.35 ± 0.2	6.74 ± 0.4	2.16 ± 0.1	7.85 ± 1.1	10.0 ± 1.2	1.90 ± 0.1	3.12 ± 0.6	1.68 ± 0.0	1.80 ± 0.0	6.39 ± 1.3	2.26 ± 0.1	6.18 ± 0.9
*Scapania gracilis*	4.98 ± 0.4	5.80 ± 0.5	2.27 ± 0.4	6.47 ± 1.2	6.62 ± 1.0	1.19 ± 0.1	3.67 ± 0.4	1.75 ± 0.2	1.94 ± 0.5	6.27 ± 1.6	3.55 ± 0.5	5.91 ± 0.4
*Frullania acicularis*	0.96 ± 0.0	5.57 ± 0.9	0.67 ± 0.1	5.73 ± 0.5	5.81 ± 0.6	1.02 ± 0.1	3.78 ± 0.3	0.68 ± 0.0	0.62 ± 0.1	3.54 ± 0.3	1.00 ± 0.5	5.77 ± 1.0
*PL—Livertorts*	2.76 ± 1.7	6.04 ± 0.8	1.70 ± 0.8	6.68 ± 1.3	7.49 ± 2.1	1.37 ± 0.4	3.52 ± 0.5	1.37 ± 0.5	1.45 ± 0.7	5.40 ± 1.8	2.27 ± 1.1	5.96 ± 0.8
*Sphagnum subnitens*	15.3 ± 0.3	19.2 ± 1.3	15.6 ± 1.4	18.6 ± 0.9	18.1 ± 0.5	8.38 ± 0.5	11.1 ± 0.8	8.14 ± 0.4	12.7 ± 0.2	16.8 ± 0.5	16.1 ± 1.8	16.8 ± 1.6
*Isothecium prolixum*	1.76 ± 0.2	4.81 ± 0.5	0.90 ± 0.1	4.89 ± 0.4	5.49 ± 0.8	1.18 ± 0.0	2.25 ± 0.3	1.00 ± 0.0	0.81 ± 0.1	3.36 ± 0.5	1.51 ± 0.3	4.77 ± 0.7
*Myur. hochstetteri*	4.73 ± 1.3	9.56 ± 0.2	1.29 ± 0.1	11.9 ± 1.4	8.87 ± 0.9	0.95 ± 0.0	2.43 ± 0.7	0.87 ± 0.1	0.93 ± 0.3	6.43 ± 0.6	2.59 ± 0.3	8.94 ± 0.7
*Thui. tamariscinum*	4.11 ± 0.5	5.31 ± 0.4	1.86 ± 0.5	5.10 ± 0.6	4.27 ± 0.4	1.28 ± 0.0	2.87 ± 0.3	1.21 ± 0.1	1.82 ± 1.0	5.88 ± 0.7	4.95 ± 0.6	5.68 ± 0.4
*PL—Mosses*	6.49 ± 5.4	9.71 ± 5.9	4.92 ± 6.4	10.1 ± 5.9	9.19 ± 5.6	2.95 ± 3.2	4.65 ± 3.8	2.81 ± 3.2	4.08 ± 5.2	8.12 ± 5.3	6.29 ± 6.0	9.06 ± 5.0
PL Total	4.89 ± 4.6	8.13 ± 4.8	3.54 ± 5.1	8.64 ± 4.8	8.46 ± 4.5	2.27 ± 2.6	4.17 ± 2.9	2.19 ± 2.5	2.95 ± 4.1	6.95 ± 4.4	4.57 ± 5.0	7.73 ± 4.0
Serra de Santa Bárbara (SB))												
*Bazzania azorica*	9.28 ± 0.5	10.3 ± 1.0	6.39 ± 0.2	8.46 ± 0.7	7.09 ± 1.3	4.32 ± 0.4	4.63 ± 0.4	2.91 ± 0.0	5.66 ± 0.1	10.3 ± 0.3	5.52 ± 0.2	9.40 ± 0.6
*Herbertus azoricus*	6.50 ± 1.7	6.33 ± 1.5	1.35 ± 0.1	4.60 ± 0.4	5.24 ± 0.5	3.65 ± 0.9	2.66 ± 0.4	1.30 ± 0.1	2.86 ± 0.6	5.32 ± 0.9	2.33 ± 0.2	4.81 ± 0.2
*Lepidozia cupressina*	10.7 ± 0.4	9.80 ± 0.4	7.15 ± 0.5	9.07 ± 0.2	5.86 ± 0.4	5.95 ± 1.0	7.27 ± 0.6	2.19 ± 0.2	6.58 ± 0.5	12.4 ± 1.1	6.49 ± 0.4	10.2 ± 0.9
*Plagiochila bifaria*	6.77 ± 1.6	6.17 ± 0.9	2.20 ± 0.7	5.24 ± 0.8	6.46 ± 1.0	5.80 ± 0.9	5.48 ± 1.3	2.42 ± 0.1		5.62 ± 0.7	2.71 ± 0.2	6.85 ± 0.7
*Scapania gracilis*	8.14 ± 1.3	6.21 ± 0.4	5.38 ± 0.8	7.79 ± 1.2	8.34 ± 1.8	3.32 ± 0.8	7.05 ± 1.2	2.22 ± 0.0	4.00 ± 0.7	9.48 ± 1.8	3.58 ± 0.1	7.85 ± 1.1
*Frullania acicularis*	7.76 ± 1.4	5.72 ± 0.7	1.20 ± 0.1	5.15 ± 0.7	8.08 ± 0.7	6.26 ± 1.3	4.14 ± 0.5	1.14 ± 0.1	1.91 ± 0.1	4.74 ± 0.4	2.08 ± 0.3	6.95 ± 0.8
*SB—Livertorts*	8.19 ± 1.8	7.42 ± 2.1	3.95 ± 2.5	6.72 ± 1.9	6.85 ± 1.5	4.88 ± 1.4	5.20 ± 1.8	2.03 ± 0.6	4.20 ± 1.8	7.97 ± 3.1	3.79 ± 1.7	7.68 ± 1.9
*Sphagnum subnitens*	17.0 ± 1.1	18.9 ± 1.0	13.2 ± 0.4	18.3 ± 1.3	16.9 ± 1.3	10.7 ± 0.8	15.2 ± 0.6	10.5 ± 0.5	14.0 ± 1.2	16.7 ± 1.0	14.1 ± 0.2	18.4 ± 0.5
*Polytri. commune*	3.21 ± 0.2	2.88 ± 0.2	2.55 ± 0.2	3.08 ± 0.2	3.03 ± 0.2	3.52 ± 0.5	2.68 ± 0.1	2.44 ± 0.1	3.04 ± 0.4	2.87 ± 0.4	2.58 ± 0.3	3.10 ± 0.2
*Campylopus shawii*	6.89 ± 0.7	5.43 ± 0.3	5.22 ± 0.3	4.91 ± 0.6	4.95 ± 0.3	4.20 ± 0.6	5.03 ± 0.5	1.91 ± 0.3	4.50 ± 0.3	7.27 ± 1.1	4.81 ± 0.2	6.57 ± 0.4
*SB—Mosses*	9.05 ± 6.1	9.09 ± 7.3	6.98 ± 4.7	8.77 ± 7.1	8.30 ± 6.4	6.14 ± 3.4	7.63 ± 5.6	4.96 ± 4.1	7.18 ± 5.1	8.93 ± 6.0	7.15 ± 5.1	9.37 ± 6.8
SB Total	8.47 ± 3.8	7.98 ± 4.5	4.96 ± 3.6	7.40 ± 4.4	7.33 ± 3.9	5.30 ± 2.3	6.01 ± 3.7	3.01 ± 2.7	5.32 ± 3.7	8.29 ± 4.2	4.91 ± 3.6	8.24 ± 4.2

## Appendix B

**Table A2:** Mean and standard deviation of the relative water content (RWC; %) for all species observed at three study sites (FS: Farol da Serreta; PL: Pico da Lagoínha; SB: Serra de Santa Bárbara) on Terceira Island, Azores. The data span one year, with measurements taken monthly. The species are categorized as liverworts (L) and mosses (M). Each data point is based on five replicates per species per month.

RWC	01_Jan	02_Feb	03_Mar	04_Apr	05_May	06_Jun	07_Jul	08_Aug	09_Sep	10_Oct	11_Nov	12_Dec
Farol da Serreta (FS)												
*Frullania acicularis*	6.72 ± 0.7	20.27 ± 1.3	3.33 ± 0.4	17.72 ± 2.6	22.36 ± 3.2	3.39 ± 0.2	3.01 ± 0.3	3.79 ± 0.4	3.49 ± 0.7	17.66 ± 2.1	7.94 ± 1.0	25.98 ± 5.1
*FS—Livertorts*	6.72 ± 0.7	20.27 ± 1.3	3.33 ± 0.4	17.72 ± 2.6	22.36 ± 3.2	3.39 ± 0.2	3.01 ± 0.3	3.79 ± 0.4	3.49 ± 0.7	17.66 ± 2.1	7.94 ± 1.0	25.98 ± 5.1
Campy. brevipilus	9.12 ± 3.0	18.70 ± 3.2	2.25 ± 0.3	22.11 ± 5.0	16.11 ± 2.1	1.93 ± 0.2	2.02 ± 0.7	1.65 ± 0.2	4.77 ± 1.4	19.85 ± 2.8	11.17 ± 2.1	19.57 ± 2.3
*Trich. brachydontium*	4.81 ± 0.5	13.55 ± 2.1	3.03 ± 0.2	8.76 ± 2.6	9.64 ± 1.4	2.40 ± 0.2	2.67 ± 0.5	2.27 ± 0.3	3.97 ± 1.8	9.92 ± 1.8	7.53 ± 1.1	13.54 ± 2.5
*FS—Mosses*	6.96 ± 3.0	16.13 ± 3.7	2.64 ± 0.5	15.44 ± 8.0	12.88 ± 3.8	2.17 ± 0.3	2.35 ± 0.7	1.96 ± 0.4	4.37 ± 1.6	14.88 ± 5.7	9.35 ± 2.5	16.55 ± 3.9
FS Total	6.88 ± 2.5	17.51 ± 3.7	2.87 ± 0.6	16.20 ± 6.6	16.04 ± 5.8	2.57 ± 0.7	2.57 ± 0.7	2.57 ± 1.0	4.08 ± 1.4	15.81 ± 4.9	8.88 ± 2.2	19.70 ± 6.2
Pico da Lagoínha (PL)												
*Plagiochila bifaria*	21.44 ± 1.6	61.60 ± 4.0	19.73 ± 0.7	71.75 ± 9.9	90.86 ± 9.7	17.36 ± 1.0	28.53 ± 5.3	15.30 ± 0.2	16.41 ± 0.4	58.3 ± 12.0	20.69 ± 1.2	56.50 ± 8.3
*Scapania gracilis*	46.64 ± 3.7	54.25 ± 5.0	21.21 ± 3.7	60.6 ± 11.4	61.96 ± 9.7	11.14 ± 0.7	34.31 ± 3.6	16.36 ± 2.1	18.12 ± 4.7	58.7 ± 15.1	33.24 ± 4.6	55.31 ± 3.7
*Frullania acicularis*	7.76 ± 0.4	44.98 ± 7.4	5.43 ± 0.6	46.26 ± 3.7	46.96 ± 5.1	8.23 ± 0.9	30.52 ± 2.7	5.52 ± 0.2	5.03 ± 0.6	28.56 ± 2.6	8.11 ± 3.8	46.63 ± 8.1
*PL—Livertorts*	25.3 ± 16.8	53.61 ± 8.8	15.46 ± 7.6	59.5 ± 13.6	66.6 ± 20.4	12.24 ± 4.0	31.12 ± 4.5	12.39 ± 5.2	13.19 ± 6.5	48.5 ± 18.0	20.7 ± 11.1	52.81 ± 8.0
*Sphagnum subnitens*	29.63 ± 0.6	37.01 ± 2.5	30.18 ± 2.7	35.93 ± 1.8	35.05 ± 1.0	16.19 ± 1.0	21.36 ± 1.6	15.73 ± 0.9	24.62 ± 0.4	32.52 ± 1.0	31.11 ± 3.6	32.54 ± 3.1
*Isothecium prolixum*	31.17 ± 2.9	84.92 ± 9.1	15.89 ± 0.9	86.31 ± 6.8	92.59 ± 6.8	20.83 ± 0.7	39.71 ± 6.1	17.74 ± 0.4	14.37 ± 1.3	59.31 ± 9.3	26.61 ± 4.9	84.2 ± 12.1
*Myur. hochstetteri*	24.05 ± 6.7	48.59 ± 1.2	6.56 ± 0.6	60.32 ± 7.3	45.07 ± 4.4	4.81 ± 0.1	12.37 ± 3.6	4.41 ± 0.3	4.74 ± 1.5	32.64 ± 3.1	13.14 ± 1.3	45.44 ± 3.7
*Thui. tamariscinum*	30.57 ± 4.0	39.44 ± 3.2	13.82 ± 3.4	37.88 ± 4.7	31.74 ± 2.8	9.49 ± 0.3	21.29 ± 2.0	9.01 ± 0.7	13.54 ± 7.3	43.67 ± 5.2	36.77 ± 4.3	42.23 ± 2.6
*PL—Mosses*	28.85 ± 4.8	52.5 ± 20.2	16.62 ± 9.0	55.1 ± 21.6	51.1 ± 25.4	12.83 ± 6.3	23.7 ± 10.8	11.72 ± 5.5	14.32 ± 8.0	42.0 ± 12.4	26.91 ± 9.6	51.1 ± 21.1
PL Total	27.3 ± 11.5	53.0 ± 16.2	16.12 ± 8.4	57.0 ± 18.5	57.8 ± 24.3	12.58 ± 5.4	26.87 ± 9.3	12.01 ± 5.3	13.83 ± 7.3	44.8 ± 15.1	24.2 ± 10.6	51.8 ± 16.6
Serra de Santa Bárbara (SB)												
*Bazzania azorica*	99.95 ± 0.1	100.0 ± 0.0	73.35 ± 2.2	95.27 ± 5.4	79.9 ± 11.6	49.57 ± 4.3	53.11 ± 4.1	33.45 ± 0.5	64.96 ± 1.0	100.0 ± 0.0	63.35 ± 2.8	100.0 ± 0.0
*Herbertus azoricus*	89.9 ± 10.3	87.5 ± 12.0	20.41 ± 2.2	69.60 ± 5.6	79.40 ± 7.9	55.3 ± 14.3	40.33 ± 5.3	19.66 ± 1.2	43.37 ± 9.3	80.6 ± 14.1	35.30 ± 3.2	72.82 ± 3.4
*Lepidozia cupressina*	74.11 ± 2.5	68.18 ± 2.7	49.77 ± 3.6	63.07 ± 1.2	40.79 ± 2.9	41.38 ± 7.1	50.59 ± 4.0	15.23 ± 1.2	45.75 ± 3.6	86.10 ± 7.8	45.17 ± 2.6	70.91 ± 6.2
*Plagiochila bifaria*	61.9 ± 14.9	56.38 ± 8.1	20.10 ± 6.8	47.87 ± 7.5	59.03 ± 9.4	52.99 ± 7.9	50.1 ± 11.8	22.08 ± 1.2		51.37 ± 6.7	24.80 ± 1.5	62.59 ± 6.3
*Scapania gracilis*	76.2 ± 12.4	58.15 ± 3.7	50.34 ± 7.6	72.9 ± 11.3	78.1 ± 17.0	31.07 ± 7.4	65.9 ± 11.6	20.78 ± 0.4	37.47 ± 6.3	87.6 ± 15.9	33.50 ± 1.1	73.5 ± 10.4
*Frullania acicularis*	62. 7 ± 11.0	46.24 ± 5.9	9.72 ± 0.6	41.59 ± 5.9	65.31 ± 5.3	50.6 ± 10.2	33.46 ± 4.2	9.18 ± 0.9	15.45 ± 1.1	38.31 ± 3.6	16.77 ± 2.7	56.17 ± 6.1
*SB—Livertorts*	77.5 ± 16.7	69.4 ± 20.0	37.3 ± 23.0	65.1 ± 18.9	67.1 ± 17.0	46.8 ± 11.8	48.9 ± 12.5	20.06 ± 7.5	41.4 ± 17.0	74.0 ± 23.8	36.5 ± 15.3	72.7 ± 15.0
*Sphagnum subnitens*	32.93 ± 2.1	36.60 ± 2.0	25.46 ± 0.8	35.38 ± 2.4	32.71 ± 2.5	20.66 ± 1.5	29.34 ± 1.1	20.35 ± 1.0	27.07 ± 2.4	32.20 ± 1.9	27.15 ± 0.4	35.60 ± 1.0
*Polytri. commune*	63.42 ± 4.1	57.01 ± 3.0	50.35 ± 4.5	60.86 ± 3.7	59.98 ± 3.2	69.62 ± 9.4	52.95 ± 1.9	48.27 ± 2.7	60.20 ± 8.6	56.71 ± 7.5	51.05 ± 5.0	61.38 ± 4.0
*Campylopus shawii*	44.64 ± 4.3	35.20 ± 2.2	33.80 ± 2.1	31.81 ± 4.2	32.05 ± 2.1	27.20 ± 3.8	32.59 ± 3.1	12.36 ± 1.7	29.17 ± 2.1	47.10 ± 7.1	31.18 ± 1.6	42.58 ± 2.8
*SB—Mosses*	47.0 ± 13.4	42.9 ± 10.6	36.5 ± 11.0	42.7 ± 13.8	41.6 ± 13.7	39.2 ± 23.1	38.3 ± 11.0	27.0 ± 16.0	38.8 ± 16.4	45.3 ± 11.8	36.5 ± 11.2	46.5 ± 11.6
SB Total	67.3 ± 21.3	60.6 ± 21.4	37.0 ± 19.7	57.6 ± 20.2	58.6 ± 20.0	44.3 ± 16.6	45.4 ± 12.9	22.4 ± 11.4	40.4 ± 16.6	64.5 ± 24.6	36.5 ± 13.9	63.9 ± 18.6

## Appendix C

**Table A3:** Detailed climate data: precipitation average (mm/month), temperature minimum and maximum (°C), relative humidity minimum and maximum (%), and vapor pressure deficit average (Pa) along 12 months in each of the three studied sites (FS: Farol da Serreta; PL: Pico da Lagoínha; SSB: Serra de Santa Bárbara) of Terceira Island (Azores). Except VPD, which was calculated, data were obtained from CLIMAAT.

Climate Variables	01_Jan	02_Feb	03_Mar	04_Apr	05_May	06_Jun	07_Jul	08_Aug	09_Sep	10_Oct	11_Nov	12_Dec
Farol da Serreta (FS)												
Precipitation (mm)	137.74	127.10	108.19	95.91	52.50	49.80	34.20	52.90	90.70	123.78	138.94	140.01
Temperature—min (°C)	11.58	11.16	11.72	12.24	13.67	15.84	17.53	19.03	18.98	16.25	1409.00	12.69
Temperature—max (°C)	16.30	16.00	16.66	17.37	19.10	21.49	23.97	25.40	24.23	21.48	18.93	17.21
Relative Humidity—min (%)	83.22	83.65	82.22	83.79	83.59	84.46	83.08	82.10	81.62	81.34	80.64	83.16
Relative Humidity—max (%)	95.58	95.01	94.48	95.18	94.96	94.00	94.33	93.29	93.73	94.40	94.65	95.47
Vapor Pressure Deficit (Pa)	169.39	170.51	186.17	179.20	194.89	236.62	280.67	325.09	325.88	266.50	239.41	182.26
Pico da Lagoínha (PL)												
Precipitation (mm)	363.84	327.07	288.13	252.51	136.73	131.35	99.28	160.50	283.66	379.19	392.11	376.58
Temperature—min (°C)	6.76	6.30	6.87	7.52	9.03	11.33	13.34	14.70	14.34	11.50	9.16	7.87
Temperature—max (°C)	11.68	11.33	12.01	12.87	14.70	17.21	20.06	21.30	19.76	16.92	14.16	12.57
Relative Humidity—min (%)	97.85	97.77	96.64	97.09	96.83	95.92	93.98	93.69	95.56	96.61	97.11	98.09
Relative Humidity—max (%)	99.72	99.75	99.62	99.61	99.53	99.44	99.21	99.28	99.47	99.60	99.66	99.76
Vapor Pressure Deficit (Pa)	14.01	14.24	21.47	20.26	25.52	37.07	66.05	72.45	48.23	30.20	22.71	13.26
Serra de Santa Bárbara (SB)												
Precipitation (mm)	480.38	443.27	354.58	314.11	165.10	144.92	100.19	160.59	309.25	455.95	485.22	504.00
Temperature—min (°C)	4.85	4.36	4.87	5.54	7.05	9.37	11.17	12.56	12.41	9.55	7.24	6.00
Temperature—max (°C)	9.94	9.57	10.18	11.07	12.88	15.42	18.04	19.31	17.97	15.12	12.40	10.87
Relative Humidity—min (%)	99.93	99.91	99.93	99.94	99.95	99.96	99.98	99.96	99.93	99.92	99.92	99.92
Relative Humidity—max (%)	99.94	99.92	99.93	99.94	99.96	99.96	99.98	99.96	99.93	99.93	99.92	99.93
Vapor Pressure Deficit (Pa)	0.60	0.90	0.75	0.64	0.61	0.56	0.34	0.73	1.19	1.12	0.98	0.75
